# Malignant melanoma of the rectum: a case report

**DOI:** 10.1186/1752-1947-3-9318

**Published:** 2009-12-04

**Authors:** Sarah Liptrot, David Semeraro, Adam Ferguson, Nicholas Hurst

**Affiliations:** 1 Department of General Surgery, Derby Hospitals NHS Trust, Derby, UK

## Abstract

**Introduction:**

Anorectal melanoma represents an unusual but important presentation of rectal malignancy. There have only been a few cases reported and the optimum management for this condition is still undecided, however, prompt diagnosis is essential. We have outlined current treatment options.

**Case presentation:**

We report a case of malignant melanoma of the rectum in a 55-year-old Caucasian man presenting as an emergency with rectal bleeding. Biopsies were taken of the fleshy mass found on digital examination, which confirmed malignant melanoma. No distant metastases were found. He underwent an abdominoperineal resection. We report the surgical management of this rare and aggressive malignancy.

**Conclusion:**

Treatment options for this condition are divergent. Surgical management varies from wide local excision to abdominoperineal resection. Clinical awareness in both medical and surgical clinics is required for prompt diagnosis and treatment.

## Introduction

In this patient, an emergency presentation of rectal bleeding led to an unusual diagnosis. Rectal bleeding is a common presentation of rectal malignancy. An uncommon form of this is malignant melanoma, attributing to only 1% of all rectal malignancies. Due to the aggressive nature of this disease, an early diagnosis and prompt treatment are essential.

## Case presentation

A 55-year-old Caucasian man, previously fit and well, presented to the accident and emergency department following a massive rectal bleed. On admission, he was haemodynamically stable with haemoglobin at 15 g/dl. His abdomen was soft and non-tender and percussion note and bowel sounds were normal. Rectal examination revealed an anterior fleshy mass at 11-12 o'clock situated 4 cm from the anal verge and just above the anorectal angle.

When questioned, the patient said he had been bleeding intermittently for 4 months but without any pain or change in bowel habit. He was a non-smoker with an unremarkable medical history.

Rigid sigmoidoscopy demonstrated a polypoid pigmented lesion at the anorectal angle. Biopsy demonstrated malignant cells with pleomorphic nuclei and abundant melanin in the cytoplasm. Completion colonoscopy was otherwise unremarkable. Computed tomography of the thorax, abdomen and pelvis and magnetic resonance imaging of the pelvis showed well-preserved anorectal fat planes and no evidence of metastasis. Dermatological and ophthalmological examinations revealed no evidence of a cutaneous or an ocular primary lesion. His case was discussed at the melanoma and colorectal multi-disciplinary team meetings.

**Figure 1:**
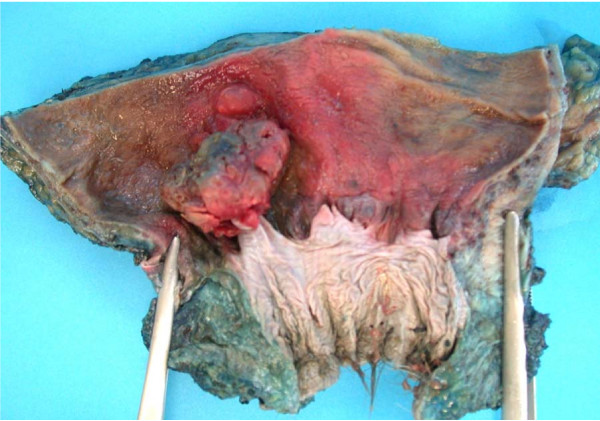
**Macroscopic image of rectal melanoma**.

Shortly after his diagnosis, the patient underwent an abdominoperineal resection (APR) without neoadjuvant treatment. He made an uncomplicated recovery and was discharged 13 days later. Immunohistochemical confirmation was obtained with cellular positivity for S100 and melan-A antigens. The malignant melanoma was completely excised with clear margins of at least 2 mm. A macroscopic image of the specimen is shown in Figure 1. At surgery, five out of seven lymph nodes were involved. He is currently being followed up by the oncology team and will be considered for chemotherapy following repeat imaging.

## Discussion

Primary anorectal melanoma is a rare disorder accounting for 1% of anorectal malignancies [1]. It is the third most common site for melanoma after the eyes and skin. It typically affects women in the fifth or sixth decade and usually presents with rectal bleeding or a change in bowel habit [2,3]. Unlike other forms, there is no association with exposure to ultraviolet light.

Lesions are most commonly found at the anorectum, followed by the anal canal and anal verge [4]. These lesions are often discounted as being benign haemorrhoids or polyps. Macroscopically, the tumours are polypoidal and pigmented while microscopically, the cells are arranged in nests with characteristic immunostaining specific for melanosome protein [5,6].

Diagnosis is often delayed and a poor prognosis is compounded by the aggressive nature of the malignancy resulting in a median survival of 24 months and 5-year survival in only 15% of cases. As a consequence, few surgical guidelines are available. Radical abdominoperineal resection may cure patients with <2 mm-wide lesions - based on the hypothesis that the disease spreads proximally via the submucosa to the mesenteric lymph nodes, it has been deemed the treatment of choice [7]. Wide local excision (WLE) has also been described as a more conservative option. Radiation is palliative in extensive tumours while combined chemotherapy is used to palliate metastatic disease. APR appears to have some effect in controlling symptoms caused by local and regional disease but has minimal impact on prognosis [8]. Prompt diagnosis and treatment are crucial to improve outcomes for those affected by this rare cancer.

## Conclusion

Malignant melanoma of the anorectum is an uncommon condition. An expeditious diagnosis and care within a mutidisclipinary team can have an important bearing on prognosis.

## Abbreviations

APR: abdominoperineal resection; WLE: wide local excision.

## Consent

Written informed consent was obtained from the patient for publication of this case report and any accompanying images. A copy of the written consent is available for review by the Editor-in-Chief of this journal.

## Competing interests

The authors declare that they have no competing interests.

## Authors' contributions

DS analysed and interpreted the data. AS and NH made substantial contributions to conception of the article and oversaw patient care. SL undertook the literature review and drafted the manuscript.
